# Diseases of the Alimentary Canal

**Published:** 1858-03

**Authors:** 


ART. III.—*Pathological and Practical Observations on Diseases of the Alimentary Canal, Œsophagus, Stomach, Cœcum, and Intestines.* By S. O. HABERSHON, M.D., London, Fellow of the Royal College of Physicians; Assistant Physician to Guy's Hospital, and Lecturer on Materia Medica and Therapeutics; Late Demonstrator of Morbid Anatomy, and Curator of the Museum at Guy's, etc. etc. London, 1857.

IF monograph writing, in medicine, be, like differentiation in physiology, a mark of advancing development, we are, surely, on the ascending grade. Time was, when the library of the practitioner need not contain other than a few treatises on "Practice," "Surgery," "Inflammation," "Fevers," etc., with, if he were learned, the writings of the "early fathers" in medicine, and of the English, French, Italian, and German classics: all a moderate number of volumes. Now, although the scope of systematic treatises is widened, and their desiderata not altogether supplied; although we have not yet (the labors of Billing, Henle, Chomel, Simon, Williams, and Stillé not forgotten) a complete Text-Book on the Principles of Medicine, yet an annual harvest of special essays fills our shelves and our minds with facts in all departments. Undoubtedly, they are all welcome; especially those proceeding from real laborers in the field, such as Stokes, Budd, Handfield Jones, Chambers, and others in Great Britain, or Gerhard, Bartlett, La Roche, Flint, and others with us.

The writer of this volume "On the Alimentary Canal," is evidently one of the *workers* in medicine and pathology. Several of his papers have already appeared in the Guy's Hospital Reports; they are here extended and amplified, so as to constitute a volume of nearly four hundred pages, upon all the important diseases of the stomach and bowels. The volume does not exhaust the subject; it ought not to be expected to do so; but it contains chiefly the record of closely followed experience, with only occasional reference to the observations of others. The writer has so devotedly adhered to the scalpel and microscope as to have allowed himself, we should judge, less culture in the use of the pen; since we meet with occasional awkwardness, even incorrectness, of style, and a total absence of fluidity in diction.

We are glad to meet, in the Preface, with a suggestion in regard to the *Dynamics* of pathology; whose almost total neglect, in ordinary pathological works, is the more remarkable, since in physiology the study of *force* occupies now so large a space. Anatomy cannot, alone, interpret disease.

"Changes produced by perverted nutrition, or altered vital forces, are in many instances of such a character that no examination of the structure itself could discern the state which had been produced; as fruitless would it be to search in the nerve of a limb for the altered force which had led to spasm, as to expect to find a telegraphic message by a microscopical examination of the wire; although the structure of both had been transiently modified by the disturbance of the force they transmitted."

A brief sketch is given, in the Introduction, of the anatomy and physiology of the stomach. Remedies addressed to its morbid state are divided into—

1. Agents used to check fermentative or chemical action.
2. To remove offending or injurious materials, or excreta.
3. To correct or improve the secretions from the mucous membrane, or those poured into the canal.
4. To affect the muscular coat and its movements.
5. To alter the state of the circulation, and vessels or absorbents.
6. To act on the abdominal sympathetic.

An important, even if trite, remark is here made, in regard to the coincidence and connection of different diseases.

"It is far from frequent to find that a patient has died free from all disease, except the one which has been the immediate cause of death; it is, indeed, the exception to find such a case. It may be, that an acute inflammation of the lungs has led to fatal results, whilst chronic disease may have been going on in the abdomen, the heart, or the brain, perhaps quite independently, but having an important influence on the curative or non-curative condition of the disease: chronic disease creeps along with unobserved step till some acute affection proves fatal. This relation of disease is worthy of our consideration in studying the affections of the alimentary canal; and we may find that the diseased conditions arrange themselves in the following manner:—

"1. They take place simultaneously in the same body, without any connection; mere coincidents.

"2. The connection may be that of different manifestations of the same disease in its progressive action, rather than a really different diseased condition.

"3. One disease may have important modifying or predisposing influence upon another.

"4. Several organs may be affected simultaneously by one exciting cause.

"5. One disease may be antagonistic of another.

"6. Other diseases, or abnormal conditions, are conservative the one to the other."—P. 9.

The last category might have been omitted; as the examples given under it, of adhesions preventing perforation by ulcers of the stomach, ileum, or gall-duct, ought rather to be considered as physiological than morbid.

Chapter II. is devoted to Disease of the Œsophagus. The causes of *dysphagia* are very varied; it resulting as follows:—

I. From disease of the tonsils or palate.

II. From diffused inflammation of the cellular tissue of the pharynx or œsophagus, or from local suppuration, sometimes in connection with disease of the spine.

III. From disease of the laryngeal cartilages, or epiglottis.

IV. From functional or spasmodic stricture of the œsophagus or pharynx, as in hysteria, hydrophobia, etc.

V. From paralysis of the muscles.

VI. From acute inflammation of the mucous membrane.

VII. From mechanical injury or poisons.

VIII. From structural obstruction to the œsophagus, as—

1. Contraction.
2. Ulcerations, sometimes communicating with the larynx.
3. Cancerous disease.
4. Obstruction from the pressure of aneurismal or other tumors.

Two cases, both fatal, are narrated, of *inflammation of the cellular tissue of the neck;* which, associated with pyœmia, or with erysipelas, is either diffused or attended with abscess.

"The latter produces sudden and urgent dyspepsia, with febrile disturbance; and on examining the throat we observe a projection from the posterior fauces sometimes on a level with the soft palate, sometimes above or below it; the diagnosis is then sufficiently evident, and, when it is possible, puncturing the abscess relieves the urgent symptoms.

"When the inflammation is diffused, the patient rapidly passes into a typhoid condition, the dysphagia becomes extreme, the respiration impeded, and, on examining the neck, we find either the erysipelatous redness of the skin, or a fullness and tenseness among the infra-hyoid muscles, impeding the free movement of the parts concerned in deglutition. The examination of the neck will generally enable us to distinguish the dyspnœa arising from this cause from that produced by disease of the larynx, or trachea, or from pressure or injury to the nerves of respiration."—P. 14.

Disease of the *epiglottis*, with ulceration, is mentioned as having been, in chronic phthisis, mistaken for organic disease of the œsophagus, in consequence of the distressing rejection of food. This condition is often relieved by inhalation of steam, or by the fumes of conium or stramonium; in less severe cases astringent gargles, or the application of a strong solution of nitrate of silver, afford comfort by diminishing the extreme sensibility, counter-irritation being also of service.

In the treatment of *spasmodic stricture of the œsophagus*, Dr. Habershon considers the use of bougies as not generally beneficial; sometimes detrimental, by tending to perpetuate and aggravate a state of spasmodic irritation and contraction,—unless we can in this way introduce nourishing food into the stomach. Morbid appearances in the *pharynx* were noted in the autopsy of a case of hydrophobia, in Guy's Hospital.

"The organ appeared more than twice its natural capacity; the constrictor muscles retracted to the utmost; the fauces exceedingly large, from the rigid contraction of the soft palate; and every part appeared expanded to the utmost. The mucous membrane was injected, and covered with some mucus. The œsophagus, also, was contracted; the lungs entirely congested; the other viscera healthy; but there was emphysema of the neck."

We have seen this emphysema of the anterior cervical region, in a case of hydrophobia, in a boy eight years of age. What is its explanation?

In the treatment of Dr. Habershon's case, it may be mentioned, by the way, that *cannabis indica* was employed, ten grains at a time, by enema, without obvious result.

*Inflammation of the mucous membrane of the œsophagus* has not been recognized by the author after death, except in one case of diptheritic gastro-enteritis, in which the pharynx and tonsils were covered with a white film (torula) spread upon an injected mucous membrane. In the management of apthous inflammation, Dr. Habershon confirms the reports of Drs. Hunt, Golding Bird, and others, upon the value of the chlorate of potassa.

*Destruction of the mucous membrane by mechanical or chemical agents*, if it do not destroy life by its primary action, is apt to be attended by inflammatory thickening and contraction, producing annular constriction of the œsophagus. The injury done by corrosive acids, or by boiling water, to this canal and to the stomach, is more often the cause of death than the harm effected in the larynx. A case is related which was fatal on the eleventh day, after swallowing sulphuric acid. Another survived the ingestion of an ounce of nitric acid for three months, dying from the secondary effects. An instance of recovery is also mentioned, after swallowing a mouthful of nitric acid. We have met with one equally remarkable, in which an eager drink of commercial oil of vitriol was taken by a boy, by mistake for porter. The symptoms were those of intense dyspnœa and aphagia, with cough, vomiting, and fever, lasting several days, but followed by recovery, without secondary symptoms.

*Annular constriction of the œsophagus* is described as resulting from the effusion of fibrinous material into the submucous cellular tissue—this tissue contracting and becoming exceedingly dense. The tube above dilates, and, the obstruction increasing, at last passage of food becomes impossible. Unless enlightened by the history of the case, Dr. Habershon knows of no direct symptom by which annular stricture can be distinguished from so-called cancerous disease.

*Ulceration of the œsophagus*, producing death by perforating the trachea, is illustrated by several specimens in the Museum at Guy's. The cases were not cancerous; the main symptom had been difficulty, at last impossibility, of deglutition: the attempt producing urgent dyspnœa, food being forcibly ejected through the nares. An interesting case is quoted from the "Pathological Transactions" for 1852:—

"A young woman, æt. twenty-five, had ulceration of the œsophagus, which extended into the pericardium, and led to sudden syncope and death. For three months she had nausea, dysphagia, occasional vomiting, and pain at the top of the sternum and at the epigastrium. Solids were swallowed with much difficulty. There was found, after death, simple ulceration, without contraction; the ulcer had extended from the bifurcation of the trachea nearly to the diaphragm, and had perforated the pericardium. No other organ was diseased."

Dr. Haberson remarks, that many persons complain of pain at the upper part of the sternum, on swallowing, in whom no trace of pressure or aneurism can be found—the symptom disappearing under the use of tonics, sometimes with iodide of potassium. The idea of cancerous growth being precluded, slight ulceration of the œsophageal mucous membrane was inferred.

The emaciation, dysphagia, and distress, in severe ulceration of the œsophagus, are identical with those connected with cancerous disease; they occur usually, however, in younger persons. The treatment of the ulceration is exceedingly unsatisfactory; the administration of nutrient enemata prolongs life only for a short time. The operation of œsophagotomy is a very difficult one, and, in many cases, if performed, would be ineffective—the disease being at the root of the lung, or behind the first bone of the sternum. The propriety of forming a gastric fistula in such cases is worthy of serious consideration: it appears warrantable, when it would afford a chance of life to those who have otherwise only a prospect of certain death. In the human subject, several cases of gastric fistula, accidentally produced, have been recorded, and experimenters upon animals make such openings without the production of severe peritonitis.

In *cancer of the œsophagus*,—

"There is pain at the sternum, in the back, sometimes in the upper part of the throat; dyspnœa comes on, when the trachea or bronchi become involved; the dysphagia and emaciation increase, and after six or seven months the disease proves fatal. Sometimes death occurs by inanition, the dysphagia having become complete; more frequently, by the extension of the disease to the bronchi, and setting up sloughing pneumonia, or by the pneumogastric nerves becoming destroyed; or, finally, by the ulceration extending into the trachea, and thus leading to fatal laryngitis. Loss of voice becomes a well-marked symptom only in those cases where the disease is situated at the base of the pharynx, extending into the larynx at that part. The part of the œsophagus which is most fixed by its connection with other organs is at the root of the lungs, and it is there that cancerous disease is most frequently found."—P. 29.

Hœmatemesis is sometimes a symptom of cancer of the œsophagus. In cancerous obstruction, we do not generally find much distension of the canal above the seat of the disease, the food being quickly regurgitated; while in annular obstruction after poisoning, etc., large dilatation occurs, the disease being more chronic, and the sensibility of the surface less.

*Epithelial* cancer was the form observed by Dr. Habershon in nearly all his cases. He believes that degeneration of the papillæ may account for the presence of some of the clusters of cells noticed, with greater probability than the endogenous mode of growth usually supposed. Schirrus and colloid are occasionally observed—the latter, only at the termination of the œsophagus.

The frequency of pulmonic complication was remarkable. In only one case out of thirteen did death appear to result from inanition; nor was this case free from disease of the lungs.

In seven there was pneumonia.
" two " gangrene of the lung.
" one " acute bronchitis and laryngitis.
" one " pleurisy.
" one " cancer of the lung, with great congestion.
" one " death from inanition.

Pressure on the pneumogastric nerve, or its destruction, extension of the disease to the bronchi, septic changes in the blood from the sloughing of the cancer, or the coincident existence of strumous disease in the lung—explained these complications. Of these thirteen cases, eight were men; their average age, fifty-six. Five were women; their average age, forty-three. The longest period between the commencement of dysphagia and death was fourteen months. Marked benefit was found to accrue from abstinence from solid food, or dependence, for a few days, upon nutrient enemata—the dysphagia being thus materially lessened.

The occasional coexistence of cancerous disease with "strumous pneumonia" or phthisis, was illustrated in three cases, in two of which vomicæ were observed; not actively progressing, however, at the time of death. Other analogous instances are given in the chapter on the stomach.

The *pressure of aneurismal or other tumors,* as a cause of dysphagia, and the possible occurrence of rupture of an aneurism into the œsophagus, producing sudden death, are briefly noticed and illustrated,—a caution being thrown out with regard to the use of the bougie, lest the latter accident be precipitated.

*Gastric solution* of the lower end of the œsophagus may be the cause of perforation and death. Only two cases, however, of this occurred at Guy's in three years. One was a case of fever, the other of hydrocephalus. Wilkinson King, in the "Guy's Reports," and Dr. Budd, in his "Diseases of the Stomach," have elucidated this subject. The position of the body, the development of gases in the intestines pressing upon the contents of the stomach, the non-contracted state of the œsophagus itself, are causes which may produce the passage of the gastric juice into the œsophagus. Sometimes this pressure has even forced the contents into the pharynx, whence they gravitate into the trachea and bronchi.

A case of *rupture of the œsophagus* from over-distension is given; the patient being an intemperate man, who died shortly after an immense supper.

Chapters III. and IV., comprising ninety-two pages, are occupied with Organic and Functional Diseases of the Stomach. Their consideration is preceded by some well-timed remarks upon the mode of examination of the stomach after death, and the need of caution in interpreting its microscopical appearances. In many examinations Dr. Habershon has found appearances precisely corresponding to the descriptions and drawings of Dr. Handfield Jones; but he thinks that many of these appearances may be produced by the mode of making the preparation, or by changes after death.

"I refer to wasting of the follicles, nuclear deposit around them, and the development of cysts. The gastric follicles change very rapidly, and in a short time nothing can be observed but the termination of the follicle itself upon the submucous areolar tissue, and above this an irregular aggregation of granules and nuclei. The basement membrane also rapidly becomes dissolved. . . . . . . . . The greater curvature of the stomach is in this way generally too much changed to allow us to place much dependence upon its microscopical examination; and for this reason it is evident that we have to avail ourselves of portions of membrane above the line of solution."

*Atrophy* and *fatty degeneration* are treated of with considerable minuteness. The symptoms presented in connection with the latter are, a sense of great prostration and exhaustion, with complete loss of appetite; the tongue clean; no pain, or thirst, or vomiting, but inability to take food; vomiting has sometimes taken place, but possibly from other causes. It has been observed "in phthisis, struma, exhausting suppuration, and often associated with fatty liver."

*Post-mortem solution* of the stomach by the action of the gastric juice was noticed even by John Hunter. The amount of solution depends, in part, on the amount of gastric juice in the stomach at the time of death. The action is much more rapid in summer than in the cold of winter. Dr. Pavy's remarkable experiments,* showing that the gastric juice will act upon living tissues, as the ear of a rabbit, the leg of a frog, etc., are considered by Dr. Habershon as not conclusive: we cannot see, however, the reason for this demurrer.

The action alluded to is stated by Dr. Budd to be checked by alcoholic liquors, or by medicines administered before death. It is most manifest in children, and with inflammatory disease of the brain, and is usually more marked in acute than in chronic disease.

In the treatment of *inflammation* of the stomach, becoming subacute or chronic, Dr. Habershon speaks favorably of the palliative influence of *lemon-juice;* which relieves the epigastric pain and distress.

Acute gastritis from corrosive poisoning was observed to pass to a fatal termination in many instances *without any pain* at the stomach, unless perforation had taken place. This absence of pain was very striking in a case in which death was produced by chloride of zinc.

*Ulceration* of the stomach is treated of as either "superficial," or "aphthous, or follicular." The history of these affections, and their treatment, agrees with that of the works of Drs. Budd, Chambers, and Brinton; especial reference being made to the investigations of the latter. The modes of termination of ulcer of the stomach, in fatal cases, were pointed out by Abercrombie:—as, 1. Gradual exhaustion; 2. Hemorrhage; 3. Perforation into the peritoneal cavity. Dr. Habershon adds a 4th: The production of inflammation by extension to the adjoining viscera. Some cases, however, will be protracted for many years; while in cancer, after well-marked symptoms, a year seldom elapses before death takes place.

An instance is mentioned of chronic ulceration of the stomach, extending to the diaphragm, in which the physical signs were those of pneumothorax.

In the management of gastric ulcer, the greatest stress is laid by Dr. Habershon on the management of the *food. This* being attended to, *medicine may* often be with advantage omitted. Horse-exercise, in mode-

* Guy's Reports, vol. ii. Third Series.

ration, is recommended. Does not this, however, admit of question, since the pain is so generally increased by movement?

If perforation occur, there is yet a bare possibility of recovery; the opiate treatment being the one relied upon. Dr. Hughes and Mr. Hilton have recorded, in the "Guy's Reports," a case in which perforation was supposed to have taken place, yet the patient recovered. We have seen one in which recovery occurred, after the discharge of a large amount of pus from the umbilicus.

*Sloughing of the mucous membrane* of the stomach is hardly to be met with, except as the effect of caustic poisons.

*Fibroid degeneration of the pylorus* is described, clearly, as differing both from cancer and from true hypertrophy. The fibrous deposit, or thickening, commences in the submucous cellular tissue. This obstructs the valve, and hypertrophy of the muscular coat then follows. The symptoms closely resemble those of cancerous obstruction. The causes of this affection are unknown. Intemperance has not been proved to promote it, and it occurs at various periods of life. In treatment, palliation may be obtained by the use of only fluid and unirritating food, and by such medication as is approved in the treatment of chronic ulceration—viz. the use of bismuth, conium, hydrocyanic acid, opium or morphia, creasote, alkalies, oxide or nitrate of silver. Dr. Jenner has pointed out the value of the sulphite of soda in checking the fermentative action which attends imperfect digestion. It may be given in scruple doses.

The mucous membrane of the stomach not unfrequently gives origin to *polypoid* growths; but their detection during life must be extremely difficult.

Every form of cancer is observed in the stomach, but medullary and schirrous cancer are the most frequent; epithelial, villous, colloid, and melanoid being more rare. These varieties, however, are known to pass into each other. The diagnosis of cancer is often, in the earlier stages, exceedingly obscure. Pain may be entirely absent throughout the whole case. Chronic ulceration usually occurs at an earlier period of life than cancer: in six cases of the former, the ages were 37, 32, 28, 36, 39, 25; in five of the latter, 40, 62, 65, 37, 47. From 35 to 55 is the age at which cancer, in any organ, is most frequently manifested.

Dr. Brinton has shown that males are more subject than females to cancer of the stomach, in the proportion of two to one. It is shown, also, by his statistics, that the pylorus is the most common seat of cancer; the lesser curvature and posterior surface, that of ulcer.

The evidence of cancer is most marked when the pylorus is affected, producing obstruction. Vomiting of blood is more frequently observed in connection with ulceration than with cancer.

A number of cases are given, in the work before us, to illustrate the varieties of cancerous disease of the stomach. Among these is one—considered to be intermediate between medullary and villous cancer—confirming the view of Paget, that the latter may be merely a variety of the more common form.

The conclusions arrived at upon the subject of gastric cancer are the following:—

"1. That the symptoms of cancerous disease of the stomach may be extremely slight, and the disease easily overlooked. 2. That the indications are more marked where the orifices are affected. 3. That in most cases death takes place from exhaustion or asthenia. 4. Total hemorrhage and perforation are more rare than in ulceration of the stomach. 5. That the absorption of degenerating cancer-structure sometimes leads to symptoms resembling pyœmia. 6. That some of the distressing symptoms may be alleviated; but that over-active treatment appears to hasten the fatal termination."

The IVth Chapter, upon "Functional Diseases of the Stomach," is chiefly occupied with dyspepsia; without containing anything, however, so novel in regard to it as to require our present attention. The *fermentation* of the contents of the stomach, dependent on imperfect digestion or unhealthy secretion, is distinguished, with Drs. Budd and Turnbull, into four varieties: 1. The formation of sulphuretted hydrogen by simple putrefactive decomposition. 2. The formation of carbonic acid in ordinary fermentation. 3. Lactic acid fermentation, or butyric acid. 4. The formation of sarcina ventriculi. Alcholic and acetous fermentation, with the development of sarcina, once believed to be pathognomonic of diseased pylorus, have repeatedly been noticed where no obstruction existed. Dr. Habershon has seen closely allied forms in the fluid on a healthy mucous membrane after death. A case of *infantile dyspepsia*, with sudden collapse, not fatal, is given; the symptoms being produced by firm coagulation of milk in the stomach and duodenum.

The *impeded movement of the stomach* is not sufficiently considered as a cause of dyspepsia. It is traceable in some cases of hernia; in tight lacing; and in those occupations which require a constrained position. When the stomach is placed vertically, its semi-digested contents are more readily impelled, prematurely, through the pylorus. Ascites and ovarian dropsy may also impair digestion by interfering with the movements of the stomach.

In some cases of over-distension from flatus, the muscular coat of the stomach is unable to contract, or becomes paralysed. But it must be borne in mind that this tympanitic state sometimes arises from inflammation coming on insidiously, and involving the muscular as well as the peritoneal coats, as in some instances of strumous diathesis. Dr. Habershon has seen several cases in which this took place, death resulting, without any pain from the commencement to the close. Such cases, which occur in youth as well as in middle life, might readily be mistaken for ordinary dyspepsia with flatulence.

*Hœmatemesis* originates in either of the following causes:—

1. It arises from ulceration of the stomach.
2. From congested or obstructed portal circulation.
3. From vicarious menstruation.
4. From cancerous disease.
5. From vitiated condition of the blood, as in purpura.
6. From aneurism.

Melœna is described as depending on hemorrhage into the stomach, from which the darkened blood passes down into the stools. Blood effused directly into the bowels does not assume this dark color. A case is related in which hœmatemesis and melœna were connected with cancer of the liver. Patients seldom die from simple hœmatemesis.

Chapter V. treats of the *Duodenum.* It is Dr. Habershon's opinion that there are symptoms of disease justly considered as arising from this portion of the alimentary canal; and that in some cases we may, with care, satisfactorily diagnose that it is the seat of disease. This occurs, however, seldom in isolation from affections of other parts, especially of the stomach, liver, heart, etc. After burns, the duodenum has been found greatly congested, sometimes ulcerated. Chronic congestion of the same tube is not unfrequently observed, along with pulmonary or hepatic congestion, or disease of the pylorus, whether fibroid or cancerous. The duodenum has sometimes been found, after death, filled with blood, extravasated from an artery opened by ulceration.

In jaundice, after exposure to cold, or after great intemperance, with vomiting, pain in the right hypochondrium, furred tongue, loathing of food, and diarrhœa, not only the stomach, but the duodenum may be irritated and congested, if not inflamed. In the treatment of these cases, Dr. Habershon considers mercurials to be rather detrimental than advantageous; bland nourishment, alkalies, and mucilaginous drinks will afford relief.

Violent acute inflammation of the duodenum is met with in practice only after the administration of poisons.

*Ulceration* of the duodenum must be remembered as a source either of fatal perforation or of intestinal hœmorrhage. Adhesions frequently take place between the first part of the duodenum and the gall-bladder; and, in some, ulceration extends from the gall-bladder into the duodenum, allowing the passage of calculi; and the gall-bladder is, in some cases, entirely obliterated.

*Mechanical obstruction* is more rare in the duodenum than in other parts of the intestines. It may result from large impacted gall-stones, enlarged cancerous glands, diseased pancreas, hydatids of the liver, or from foreign bodies. The most marked symptom in these cases has been the very early period at which violent bilious vomiting has supervened; the absence of distension of the abdomen is also a sign of the occlusion being high up.

Perforation of the duodenum occasionally takes place after death, from solution, by the gastric juice passing through a patulous pylorus.

*Enteritis* is considered in the VIth Chapter, under the heads of Erythematic Enteritis, or *Muco-enteritis*, and Severe Acute Enteritis. The former corresponds with the gastric fever, or infantile remittent, of writers. The latter may be quite dangerous, involving all the coats of the intestine. Its most intense form is seen when a portion of the intestine becomes strangulated.

Internal or external hernia, intussusception, or mechanical obstruction from any cause, may be confounded with simple enteritis. In internal strangulation, the pain generally comes on after sudden exertion; in internal obstruction without strangulation, we often find previous constipation, and the commencement of the attack is slower—the pain being sometimes slight till toward the close. In intussusception, the sudden severe pain is more like that of colic than of enteritis; and the discharge of bloody mucus is often observed, as pointed out by Mr. Gorham.*

The diagnosis between *muco-enteritis* and *hydrocephalus* is not always easy. In cerebral distubance accompanying the former, however, we have, commonly, emaciation, diarrhœa, a half-closed eye, and collapsed fontanelle; and the earliest symptoms are those not of cerebral, but abdominal disease.

In acute enteritis, leeching, and bleeding from the arm, are spoken of as advantageous. The author is, therefore, not yet *Bennett-ized.* The chlorate of potassa is highly commended in the muco-enteritis of children.

Chapter VII. is devoted to *Strumous Disease* of the Alimentary Canal, including that of the Mesenteric Glands and Peritoneum. In one hundred cases of phthisis, only thirteen had the intestines healthy. It is only in "pneumonic phthisis," or galloping consumption, that the alimentary canal escapes the effects of the constitutional disease. The bowel affection, however, does not necessarily keep pace with that of the lungs. Matter of much interest upon this subject might be extracted, did our space allow it. In the diarrhœa of phthisis, Dr. Habershon recommends the injection of borax or charcoal, with barley-water.

*Diseases of the Cœcum and Appendix* appear in Chapter VIII. Contrary to the experience of Tiedemann and Gmelin, Dr. Habershon has found the secretion of the cœcum *alkaline* in reaction. There is, to his

* Guy's Reports, 1858, p. 30.

mind, no warrant for the statement that the cœcum constitutes a second stomach, in which digestion is completed.

The appendix he considers to be an elongated gland, of a very simple character, analogous to the pancreatic cœca of the intestine of the fish; so far as is at present known, its secretion is ordinary mucus. Its action appears to be, to prevent adhesion of fœces to the parietes of the intestines.

*Ulceration* commences more frequently in the *appendix* than in the cœcum itself. The appendix may also be elongated, atrophied, dilated, or filled with concretions. The latter may be either—1. Extraneous, such as nails, pins, stones of fruit, bristles of a tooth-brush, entozoa, or, most frequently, fœces. 2. Formed in the appendix; as concrete or albuminous mucous. 3. Calculi, generally with a fœcal or foreign nucleus. Many of the so-called cherry-stones are of this character. However produced, such concretions may lead to very serious results, by exciting irritation, ulceration, perforation, or suppurative inflammatory action, ending in fœcal abscess. In phthisis it is very common to find ulceration of the appendix cœci.

Important points in the diagnosis of cœcal disease are elucidated, for which the reader must be referred to the work itself. There are few matters more trying to the diagnostic acumen of the practitioner. One paragraph may be quoted:—

"The neuralgic pain connected with urino-genital disease is not accompanied by the tenderness or other symptoms of intestinal affection. It is, however, sometimes difficult to distinguish inflammatory disease about the right ovary from cœcal disease. There may be, in both, excessive tenderness, febrile excitement, constipation, severe pain in the lower part of the iliac fossa. The symptoms which will serve to guide us are, that the ovarian disease comes on with irregular menstruation or with sudden cessation of that flux, and that the pain is situated lower down in the hypogastric region;—in some cases even, observers have believed that they have felt the swollen ovary. Dr. Barlow records a case in which peritonitis of such a severe character was set up around an inflamed ovary that the patient succumbed. In cancerous disease of the cœcum, which sometimes occurs in young subjects, it is almost impossible, unless there be indication of cancerous disease in other parts, rightly to diagnose its character. These are, however, rare cases. In strumous peritonitis, the disease is not confined to one part of the abdomen; but in severe cases, the intestines are so completely united by peritoneal adhesion as to move *en masse*."—P. 208.

When collapse and tympanites evince perforation of the intestine, rest, the avoidance of aperients, and the use of opium, perhaps with calomel, are advised. The detection after death of foreign substances in the appendix, without apparent injury having resulted, is not uncommon. Dr. Habershon has found there a pin, with its head downward and its point extending into the coats, half surrounded by fibrous membrane; in another instance an iron nail, etc.

A curious case is given, in which inflammation of the cœcum simulated *hip-joint disease;* the right leg being *rigidly* flexed at the thigh, in consequence of pain; and relaxing after leeching and poulticing the abdomen. Two instances are recorded, also, of protrusion of the appendix into the inguinal canal.

*Diarrhœa* is the subject of the IXth Chapter; being classified into diarrhœa *crapulosa, serosa, mucosa, biliosa,* and *dysenterica.* The causes of this affection are—1. Cold and wet. 2. Improper food. 3. Inanition or exhaustion. 4. Epidemic influence. 5. Endemic influences, such as effluvia from drains, etc. 6. Excessive secretion of bile, or of the glandular fluids. 7. Strumous disorder of the intestine or mesenteric glands. 8. Œdema or congestion of the mucous membrane, as in Bright's disease. 9. Mental agitation or fright. 10. Ulceration or cancerous disease of the large or small intestine.

*Colitis* and *Dysentery* are considered at length in Chapter X. The author does not admit the correctness of Dr. Parke's view, that, in true dysentery, ulceration, or, as Dr. Baly describes it, sloughing, is always present. Death may, although rarely, take place without destruction of the mucous membrane in any part. An elaborate history is given of the changes ordinarily presented in the dysenteric bowel.

The *sequelæ* of dysentery, when not fatal during the attack, are—1. Perforation of the intestine, and fatal peritonitis. 2. Fæcal abscess. 3. Gradual exhaustion from destruction of the mucous membrane. 4. Constipation, from contraction of cicatrices, leading to very troublesome and irregular condition of the bowels, and sometimes fatal obstruction. 5. Pyœmia, and suppuration in the substance of the liver, from the absorption of pus, as described by Dr. Budd. This last result Dr. Habershon has observed only once at Guy's, in simple English dysentery. It is more common in the tropical form. The author does not positively adopt the opinion that dysentery is contagious. He denies that it is a blood-disease, or of constitutional origin, any more than cancrum oris, or ulcerative stomatitis. This may be true of "English dysentery." We cannot, however, believe it so of the epidemic and endemic varieties, so common in many parts of this country, and elsewhere.

Besides hemorrhoids, *polypus in the rectum* and *fibro-cellular ulceration* may be mistaken for dysentery. In the treatment of the latter disease the measures most generally approved are described and recommended. From the experience of a number of severe cases, it is inferred that, in the worst cases of dysentery, astringents and opiates are ineffective; that injections and demulcent remedies afford considerable relief, and in mild cases will be sufficient, but are inferior in efficacy to astringents and opium; that rest, even in mild cases, is important; and that in the cases alluded to, as far as could be judged, mercurial preparations would have been injurious.

Under the title of "*Typhoid Disease of the Intestine,*" we find described, in a short chapter, the usual lesions of typhoid fever. A case is mentioned, in which the patient was so little enfeebled as to walk about during the whole of the attack; yet he died of perforation of the intestine on the twenty-third day. Dr. Habershon strongly urges caution in the return to solid food or active movement; considering that by such precaution many lives may be saved.

Chapter XII. follows, upon *Colic.* The subdivisions of Dr. Copland are adopted, and a fair *rèsumê* is given of the semeiology, pathology, and treatment of the different varieties.

*Constipation* is well treated of in the XIIIth Chapter. Among other interesting points, a case is quoted from Mr. Staniland (*Medical Gazette,* 1832–3) in which for five years the patient had a passage from the bowels only once in two months.

*Intestinal obstruction,* in its numerous forms, is the subject of one of the most important chapters in the book. The causes of insuperable constipation are, mainly,—

1. Bands of adhesion, producing internal strangulation.
2. Twisting of the intestine, in either of three modes described by Rokitansky.
3. Mesenteric tumors, producing constriction.
4. Intussusception.
5. Cancerous disease of the intestine.
6. Contraction of cicatrix, as after dysentery or fever.
7. Enteritis and peritonitis.
8. Impaction of fæces, or foreign bodies, gall-stones, etc.
9. Obturator or other obscure forms of hernia.
10. Prolapsus ani, and inflamed hemorrhoids.
11. Abdominal or pelvic tumors.

The sign noted by Dr. Barlow, of the diminution in the quantity of urine excreted, when the obstruction is high up, is viewed by Dr. Habershon as not certain. A manual examination of the rectum is advised in all cases. Dr. Habershon has never met with an instance of one loop of intestine becoming twisted upon another as an axis. Peritonitis was observed to occur almost universally in fatal intestinal obstruction.

A specimen is alluded to, in the museum at Guy's, consisting of the whole of the cæcum and ascending colon, which were evacuated by a patient suffering from intussusception, but who, nevertheless, *recovered.* Forty-four inches of the large intestine were passed in another case. The *cause* of intussusception is believed to be, sudden spasmodic contraction of a portion of the intestine, impelled onward into a portion less contracted or altogether flaccid.

*Cancerous disease* is especially prone to attack the termination of the sigmoid flexure, at the brim of the pelvis. Scirrhus is the most frequent form, although medullary and colloid sometimes occur.

In regard to the diagnosis of the different varieties of obstruction,—

"In *intussusception* there is generally more pain, like colic; there is discharge of bloody mucus, and a tumor is frequently felt at the affected part. In *internal strangulation* the vomiting is more severe, the onset more sudden than in cancerous disease, and frequently something is felt to have given way or slipped; there is greater resemblance to the symptoms of ordinary hernia; the small intestine is the part that is generally strangulated; and while the vomiting is more early and severe, the abdomen is less distended, and the course of the colon cannot be traced. In *impacted fæces* alone, unless some foreign body be also present, the symptoms rarely, if ever, become so urgent, and scarcely ever fatal."—Pp. 322–3.

In the treatment of obstruction of the bowels, Dr. Habershon objects to the employment of any but mild aperient medicines. Purgatives of all kinds, he considers, should be avoided; drastics tend only to aggravate the patient's sufferings, to shorten life, or to remove the remaining possibility of recovery. The administration of opium is relied on, as tending more than any other medication to the relaxation of spasm, and evacuation of the bowels. Injections, laxative and nutritive, are approved. Change of position, sometimes useful, requires caution, lest it incite to inflammation. The application of cold water or cold air, to the abdomen, has sometimes succeeded. In regard to operative interference, as practiced by Hilton and others, it is suggested by our author—1st. That the peritoneum is already inflamed, or intensely congested, and hence general peritonitis is almost certain to follow; and 2d. That there is a great difficulty in the diagnosis, and that some recover from apparently a dying condition. In Cæsar Hawkins's operations for stricture of the colon or rectum, by the production of artificial anus, out of forty-four cases, in ten, death took place within forty-eight hours; in twenty-one, within five weeks. Thirteen recovered, of whom six lived six months, and nine more than a year.

A brief chapter ensues upon *Intestinal Worms*. Dr. Habershon has found the *tricocephalus dispar* much less often than he was led to expect by other writers. In the treatment of *tape-worm*, at Guy's Hospital, turpentine and kousso have given way to the *oil of male fern*. This appears to be innocent, even in large quantity; a child, by mistake, took ℥iss of it every night for a week. No symptom except purging followed.

Chapter XVI. concludes the volume, with the discussion of the subject of *Perforation of the Intestine from without* This may proceed—

1. From strumous peritonitis.
2. From ulceration or cancer of the stomach.
3. From hydatids, or abscess of the liver.

4. From the penetration of gall-stones from the gall-bladder.
5. From abscess in the spleen, kidney, abdominal parietes, or loins.
6. From disease of the ovary.
7. From cancer, in various structures.
8. From extra-uterine fœtation.
9. From one portion of the intestine opening into another, as the appendix into the rectum.
10. From blows or external injury.

The diagnostic signs of these different affections are clearly described, and illustrated by examples. In the whole of the volume, one hundred and seventy-three cases are detailed, including every variety of abdominal disease—nearly all of them having occurred under the writer's own observation. They are judiciously selected, and briefly recorded, being illustrated, also, by three plates, prefixed to the work.

We take leave of Dr. Habershon with the conviction that his book is a decidedly useful one. Without the systematic fullness of Budd, or the vivacity of Chambers, he conveys a vast number of facts in an instructive manner. To wield the pen of elegance is an accomplishment which so ardent a pathologist may easily afford to leave to other hands.

